# MRI in radiotherapy — Is it time to rethink the current radiotherapy fixation solutions?

**DOI:** 10.1120/jacmp.v15i6.5192

**Published:** 2014-11-08

**Authors:** T. Nyholm, T. Mullaney, L.E. Olsson, K. Finnilä, B. Zackrisson

**Affiliations:** ^1^ Department of Radiation Sciences Umeå University Umeå Sweden; ^2^ Umeå Institute of Design, Umeå University Umeå Sweden; ^3^ Department of Medical Radiation Physics Lunds University Lund Sweden

To the Editor:

Radiotherapy is an important treatment modality for patients with head and neck or brain tumors. The treatment is usually performed with the patient in a restraining device (fixation) for reproducible positioning and to minimize intrafraction movement. The most commonly used methods are specially designed pillows and either thermoplastic face masks or biting blocks. The fixation equipment traditionally used in radiotherapy is, however, not compatible with state‐of‐the‐art magnetic resonance imaging (MRI). This is not due to risk of heating, mechanical force or any other common problem within the extreme environment of a MR scanner. It is purely a problem of size. The standardized pillows, masks, and bite block fixations do not fit within an MR head and neck coil. This problem has been identified by all major MR vendors. The current suggested solution is to use flexible coils around the patient's head instead of the designated head and neck coils.^1^, ^2^ This is a poor solution for several reasons. There is a reason why designated coils have been developed in the first place — they provide optimal image quality. Using the flexible MRI coils for radiation therapy may trade image quality for practicality. Additionally, modern and efficient imaging techniques, such as parallel imaging, may be hampered by the suboptimal coil design. Are the requirements on image quality less in radiotherapy compared to the diagnostic setting? The gross tumor should be delineated with high precision on anatomical images, and technically demanding functional imaging techniques are used to identify more active or radioresistant volumes. Working with flexible coils is more complicated for personnel; it takes more time to set up, and the risk for mistakes affecting imaged quality is increased.

The fixation devices used in diagnostic MRI are designed for both patient comfort and immobility and we suggest that, with small modifications, they have the potential to be used in radiation therapy setup. So instead of putting resources on solutions to adapt the MR equipment to the radiotherapy environment, compromising image quality, these resources should be spent on redesigning the radiotherapy fixations instead. Face masks have been described as a significant source of anxiety in this patient group.^(3)^ Therefore, a key requirement, besides compatibility with MR, should be the patient‐friendliness of the fixation. The introduction of MR as a tool for treatment planning and the extensive use of image‐guided radiotherapy offer a timely opportunity for innovations in this area! (Fig. 1)

**Figure 1:**
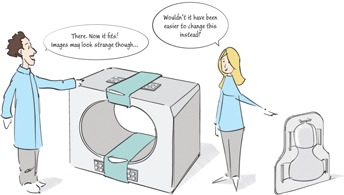
Perhaps it would be a good idea to adopt the RT fixations to the requirements from MR instead of the other way around.

## References

[1] Ahmed M , Schmidt M , Sohaib A , Kong C , Burke K , Richardson C , et al. The value of magnetic resonance imaging in target volume delineation of base of tongue tumours – a study using flexible surface coils. Radiother Oncol. 2010;94(2):161–67. 20096947 10.1016/j.radonc.2009.12.021

[2] Van der Heide UA , Houweling AC , Groenendaal G , Beets‐Tan RGH , Lambin P . Functional MRI for radiotherapy dose painting. Magn Reson Imaging. 2012;30(9):1216–23. 22770686 10.1016/j.mri.2012.04.010 PMC5134673

[3] Mullaney T , Pettersson H , Nyholm T , Stolterman E . Thinking beyond the cure : a case for human‐centered design in cancer care. Int J Des. 2012;6(3).

